# Induction of DARPP-32 by Brain-Derived Neurotrophic Factor in Striatal Neurons *In Vitro* Is Modified by Histone Deacetylase Inhibitors and Nab2

**DOI:** 10.1371/journal.pone.0076842

**Published:** 2013-10-21

**Authors:** Samira Chandwani, Serene Keilani, Maitane Ortiz-Virumbrales, Andrika Morant, Steve Bezdecny, Michelle E. Ehrlich

**Affiliations:** Departments of Neurology and Pediatrics, Mount Sinai School of Medicine, New York, New York, United States of America; National University of Singapore, Singapore

## Abstract

Neurotrophins and modifiers of chromatin acetylation and deacetylation participate in regulation of transcription during neuronal maturation and maintenance. The striatal medium spiny neuron is supported by cortically-derived brain derived neurotrophic factor and is the most vulnerable neuron in Huntington’s disease, in which growth factor and histone deacetylase activity are both disrupted. We examined the ability of three histone deacetylase inhibitors, trichostatin A, valproic acid and Compound 4 b, alone and combined with brain derived neurotrophic factor (BDNF), to promote phenotypic maturation of striatal medium spiny neurons *in vitro*. Exposure of these neurons to each of the three compounds led to an increase in overall histone H3 and H4 acetylation, dopamine and cyclic AMP-regulated phosphoprotein, 32 kDa (DARPP-32) mRNA and protein, and mRNA levels of other markers of medium spiny neuron maturation. We were, however, unable to prove that HDAC inhibitors directly lead to remodeling of *Ppp1r1b* chromatin. In addition, induction of DARPP-32 by brain-derived neurotrophic factor was inhibited by histone deacetylase inhibitors. Although BDNF-induced increases in pTrkB, pAkt, pERK and Egr-1 were unchanged by combined application with VPA, the increase in DARPP-32 was relatively diminished. Strikingly, the NGF1A-binding protein, Nab2, was induced by BDNF, but not in the presence of VPA or TSA. Gel shift analysis showed that α-Nab2 super-shifted a band that is more prominent with extract derived from BDNF-treated neurons than with extracts from cultures treated with VPA alone or VPA plus BDNF. In addition, overexpression of Nab2 induced DARPP-32. We conclude that histone deacetylase inhibitors inhibit the induction of Nab2 by BDNF, and thereby the relative induction of DARPP-32.

## Introduction

Transcriptional regulatory mechanisms specifying neuronal subtype differentiation and gene expression remain enigmatic for most neurons in the central nervous system. The striatum, a component of the basal ganglia, is involved in motor coordination, emotion and cognition, and transcriptional regulation or dysregulation in its neurons is a feature of many prevalent neuropsychiatric diseases and their treatments. GABAergic, medium-sized spiny neurons (MSNs) are the striatal output neurons and comprise 90–95% of its total neurons [1]. Dopamine and cyclic AMP-regulated phosphoprotein, 32 kDa (DARPP-32), encoded by the *ppp1r1b* gene, is expressed in 98% of the MSNs and modulates their response to dopamine and other first messengers [2]. Although DARPP-32 is in fact expressed at a lower level throughout the forebrain, it is the most commonly used marker of the mature, post-mitotic, post-migrational MSN [3].

Brain-derived neurotrophic factor (BDNF) is a major regulator of MSN phenotype during development and in the adult, and multiple molecular mechanisms via which it regulates *ppp1r1b* transcription have been well characterized [4]–[7]. Many factors that contribute to neuronal differentiation and plasticity, including BDNF [8], lead to alterations of chromatin structure via core histone modifications, and inhibition of histone deacetylase (HDAC) activity promotes this process [9]. In most cases, histone acetyltransferases (HATs) acetylate lysine residues on the amino terminal tails of core histones (H2A, H2B, H3 and H4), thereby relaxing chromatin structure and allowing for transcriptional activation. HDACs promote chromatin condensation by removing acetyl groups and therefore usually act as transcriptional repressors. HDAC inhibitors increase the association of acetylated histones with chromatin, thereby again relaxing chromatin condensation (reviewed in [10]). Importantly, however, HDAC inhibitors activate multiple signal transduction pathways and may also directly or indirectly lead to transcriptional repression [10]–[12].

Loss of striatal, cortically-derived BDNF and aberrant histone regulation are two major factors leading to transcriptional dysregulation in Huntington’s disease (HD) [13]. A combination of BDNF and an HDAC inhibitor (HDACi) is a potential therapeutic cocktail for HD, and is already used to derive mature MSNs from iPS and ES cells [14]. We sought to determine the ability of HDAC inhibitors (HDACi) to promote expression of DARPP-32 and other markers of MSN maturation *in vitro*, either alone or in combination with BDNF. We therefore focused on HDAC inhibitors that have been evaluated in models of polyglutamine disease [15] and in the promotion of the MSN phenotype from iPS and ES cells [14]. Two classical, broad-spectrum HDAC inhibitors, trichostatin A (TSA), a hydroxamic acid, and the small carboxylate molecule, valproic acid (VPA), increase acetylation of histone H3 in HD, thereby restoring levels of some of the dysregulated transcripts [16]–[17]. Compound 4 b is a pimeloylanilide derivative that targets acetylated histone H4, and also corrects transcription and behavior abnormalities in R6/2-300Q transgenic mice [18]. VPA is utilized in almost all iPSC differentiation protocols for MSNs, but the direct effect of HDACi’s on MSNs has not been assayed. We report that treatment with all three HDAC inhibitors results in a two-to-three-fold increase in DARPP-32 protein levels in MSNs *in vitro*, but surprisingly, reduces the induction of differentiation by BDNF without interfering with any of the heretofore identified signal transduction pathways utilized by BDNF in this process. We determine that BDNF also up-regulates the NGF1A binding protein, Nab2, and that opposite effects on induction of Nab2 by VPA appear to mediate this apparent antagonistic action.

## Materials and Methods

### Cell Culture and Viral Transduction

Neurons were derived from the E15–17 embryonic striatum. The protocol for preparation and maintenance of the cultures has been previously described [3]. Additives after 1 DIV (day *in vitro*) included: Trichostatin A (Cell Signaling, Danvers, MA, USA); Compound 4 b (Calbiochem/EMD Biosciences, San Diego, CA, USA); Valproic acid (Sigma Aldrich, St. Louis, MO, USA) and BDNF (PeproTech, Rocky Hill, NJ). V5-Nab1 or GFP under the control of the CMV promoter in adenovirus (SignaGen Laboratories, Rockville, MD) were added at a multiplicity of infection of 100 after cells had attached for 1–2 h. Virus was added in fresh medium, and cells were harvested after 96 h. BDNF (10 ng/ml) was added 24 h prior to harvest.

Animals were housed and employed in research with the approval of the Mount Sinai Institutional Animal Care and Use Committee (IACUC) which specifically approved this study.

### Immunocytochemistry

Cultures were immersion fixed in 4% *para*-formaldehyde in 0.1 M phosphate buffer, pH 7.4 for 30 min and processed using rat monoclonal α-DARPP-32 antibody (Cell Signaling, Danvers, MA) (1∶1000) and anti-rabbit secondary antibody (Vector Laboratories, Burlingame, CA)(1∶400). Slides were developed with the immunoperoxidase/ABC method (VECTASTAIN Elite ABC Kit, Vector Laboratories Burlingame, CA).

### Western Blot Analysis

For analysis of non-phosporylated holoproteins, e.g. DARPP-32, cells were harvested in 20 mM Tris-HCl, pH 8.0, 137 mM NaCl, 1 mM EDTA, 1 mM Na_3_V0_4_, 5 µM ZnCl_2_, 100 mM NaF, 1 µM Pepstatin and 10% v/v Triton X-100 supplemented with 1× Mini-Complete protease inhibitors (Roche Diagnostics Corporation, Indianapolis, IN, USA) and processed as described previously [6]. For analysis of phosphoproteins, and as a control, their respective non-phosphorylated isoforms, total cellular protein was prepared as previously described [5]. Blots were developed using the Fujifilm LAS-4000 Plus Gel Documentation System and visualized using Fujifilm Image Reader LAS-4000 (Fujifilm Holdings Corporation, Tokyo, Japan). Densitometric values were obtained using Multi Gauge software for analysis (Fujifilm Lifesciences, Tokyo, Japan). Statistical analysis was conducted using Graphpad Instat software (GraphPad Software, La Jolla, CA). Results were considered significant if *p*<0.05. Antibodies used included the following: DARPP-32 (1∶1000); Phospho-ser472-Akt (1∶1000); Akt (1∶1000); phospho-p44/42 thr202/tyr 204 (1∶1000); trkB (1∶1000) (BD Biosciences, Sparks, MD, USA); phospho-TrkB (1∶1000) (Epitomics, CA, USA); histone H3 (1∶200); H4 (1∶200); acetyl Histone H3 (1∶1000); acetyl Histone H4 (1∶1000) (Millipore, Billerica, MA,); GAPDH (1∶5000) (Santa Cruz Biotechnology, Santa Cruz, CA); Bcl11b (1∶1000) (Bethyl Laboratories, Montgomery, TX); Egr-1, clone 588 (1∶2000) (Santa Cruz Biotechnology, Santa Cruz, CA); Nab1 (Santa Cruz sc-12147; 1∶1,000); Nab2 (Millipore AB15334 1∶1,000). Statistical analyses were performed with one way ANOVA and Bonferroni post-hoc tests except where indicated.

### Cytotoxicity/Viability Assay

Cytotoxicity and Viability assays were conducted using the LIVE/DEAD Viability/Cytotoxicity Kit for mammalian cells (Invitrogen, Carlsbad, CA). Microscopy was performed with an Olympus 1×51 equipped with the Xcite Series 120 apparatus for detection of fluorescence. Viable cells were detected using a standard fluorescein bypass filter (ex/em 495 nm/515 nm) and cytotoxic cells were detected using a Texas red dye filter (ex/em 495 nm/635 nm).

### Chromatin Immunoprecipitation

80–150 mg of striatal tissue from adult wild-type mice or 1.0×10^7^ NIH 3T3 cells per reaction were fixed using 1% formaldehyde in 1× PBS at RT and crosslinking was quenched with glycine (final concentration: 0.125 M). Tissue was centrifuged for 5 min at 2000×g at 4°C and pellet was washed with 1× PBS. Tissue was resuspended in 5∶1 volume of cell lysis buffer (10 mM HEPES pH 8.0, 85 mM KCl, 0.5% NP-40 supplemented with protease inhibitors) and homogenized on ice for 2–3 min using a handheld motorized pestle. Homogenized samples were centrifuged at 5000×g for 5 min at 4°C to pellet nuclei. Nuclear pellet was resuspended in 10∶1 volume of nuclear lysis buffer (1% SDS, 10 mM EDTA pH 8.0, 50 mM Tris-HCl pH 8, supplemented with protease inhibitors) and incubated on ice for 10 min. Tissue samples were sonicated 10 times, and cellular samples 6 times, using a Branson 450 sonicator (setting 3, duty cycle 50%) and centrifuged for 15 min at 14,000×g at 4°C to obtain the whole cell extract (WCE). 10 µL of WCE was reserved as input. Remaining WCE was diluted 10 times with dilution buffer (1% Triton X-100, 150 mM NaCl, 2 mM EDTA pH 8, 20 mM Tris-HCl pH 8 supplemented with protease inhibitors) and incubated overnight at 4°C with 2 µg of antibody (Acetylated Histone H3 kit, ChampionChIP Mouse ChIP-Grade antibody, SABiosciences, Frederick, MD) or IgG as a control. After 12–18 h lysates were incubated with sheep anti-rabbit IgG beads (Dyna Beads, Invitrogen, Carlsbad, CA) overnight at 4°C. Beads were then pelleted and washed 3 times with low salt lysis buffer (1% Triton X-100, 0.1% SDS, 150 mM NaCl, 2 mM EDTA pH 8, and 20 mM Tris-HCl pH 8), 2 times with high salt lysis buffer (1% Triton X-100, 0.1% SDS, 500 mM NaCl, 2 mM EDTA pH 8.0, 20 mM Tris-HCl pH 8.0), 1 time with Tris EDTA buffer (50 mM NaCl, 10 mM Tris-HCl pH 8, 1 mM EDTA pH 8). Protein–DNA complexes from immunoprecipitation (IP) samples and Input were eluted using 300 µL of elution buffer (1% SDS, 0.1 M NaHCO_3_) at 65°C for 30 min. Crosslinks were reversed overnight at 65°C. Eluted DNA was treated with RNase and incubated at 37°C for 30 min and boiled at 95°C for 15 min. Real-time quantitative polymerase chain reaction (RT-qPCR) was performed on IP samples (or on RNA samples prepared from cultured neurons) and Input using the *Ppp1r1b* +1a Primer Sequence from SA Biosciences (ChIP-qPCR Assay GPM1029882(+)01A for Mouse *Ppp1r1b*, NM_144828.1 (+) 1 kb, Frederick, MD, USA) using the AB Step One Plus Real-Time PCR System and data was analyzed using the Step One Software (Applied Biosystems, Foster City, CA, USA). Each ChIP reaction Ct value was then normalized to the Input DNA Ct value as follows: deltaCt [normalized ChIP] = (Ct [ChIP] – (Ct [Input] – Log2 (Input Dilution Factor). The percentage of DNA recovered from each immunoprecipitation reaction relative to the starting DNA was calculated as % Input as follows: % Input = 2 (−deltaCt [normalized ChIP]).

### Quantitative Reverse Transcription-PCR Analysis

RT-qPCR was performed with TaqMan kits (Invitrogen) as described previously [19]. Quantitative PCR following ChIP was performed by SYBR® Green based real-time PCR using RT^2^ SYBR® Green qPCR Master Mixes (SABiosciences, # PA-012) according to the manufacturer’s instructions. PCR products were detected by ethidium bromide on a Fujifilm LAS-3000 developer following electrophoresis on 1% Tris borate-EDTA agarose gel for 1 hr at 60 V. For data analysis, the threshold cycles (Ct) of all replicates were averaged after dropping reactions with PCR inhibitors or a varying slope as determined from the melt and amplification curves.

### Electrophoretic Mobility Shift Assay (EMSA) and Super-shift Assays

Double-stranded oligonucleotides (Integrated DNA technologies, USA) were prepared in annealing buffer [20 mM Tris, 10 mM MgCl2, 50 mM NaCl, 1 mM DTT] to a final concentration of 5 µM. The double-stranded DNA fragments, aka H10 [7] (5′-AGCCGCCCACACTGTTCCTTTCC-3′) were labeled with ATP [γ-^32^P] and T4 polynucleotide kinase, and purified with Illustra Microspin G-25 columns (GE Healthcare). Each EMSA reaction (25–30 µl) contained 5–10 µg nuclear proteins, 2 µg Poly (dI-dC), 1 pmole DNA probe in 1× binding buffer [10 mM HEPES pH = 7.9, 30 mM KCl, 1.2% glycerol, 0.5 mM DTT, 5 mM MgCl2, 0.2 mM PMSF, 0.1 mM EDTA pH = 8]. The protein-DNA complexes were separated on native 6% polyacrylamide gels in 0.5X Tris borate/EDTA running buffer at 200 V for 2 hours. The gels were dried and exposed on phosphor-screens (GE Healthcare) using the Typhoon- Trio (GE Healthcare). For super-shift assays, 2 µg of α-Nab2 antibody were added to each reaction and incubated for 30 minutes at 4°C prior to, or after, the addition of the ^32^P -labeled H10 probe.

## Results

### HDAC Inhibitors Increase Levels of DARPP-32 and Calbindin, and Decrease bcl11b Protein in MSNs *in vitro*

**Figure 1:**
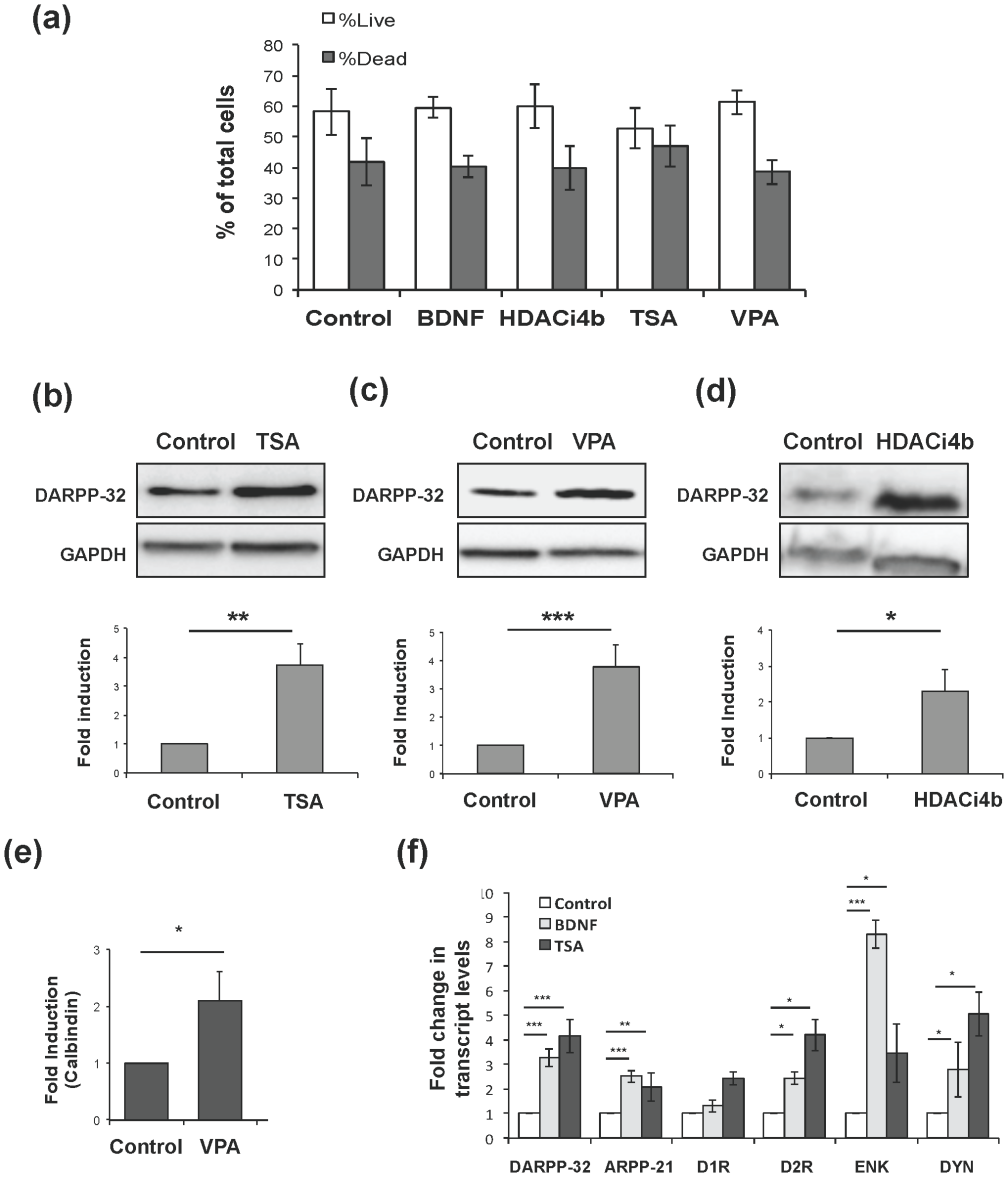
Trichostatin A, valproic acid and HDACi 4b up-regulate markers of the differentiated MSN but do not increase survival of striatal neurons *in vitro*. (**a**) Over 2,000 neurons were counted for each condition, derived from 3 separate platings. Untreated control, BDNF (10 ng/ml), HDACi 4b (5 µM), trichostatin A (10 nM) and valproic acid (3 mM), p = 0.94, one-way ANOVA. (**b–d**) Primary striatal neurons were treated with (b) TSA (10 nM), (c) VPA (3 mM), or (d) HDACi 4b (5 µM) for 24 h, each of which increased the level of DARPP-32 protein. Representative of 3 platings, each performed in duplicate. (**e**) Calbindin is increased after 24 h of treatment with VPA, (**f**) Transcript levels of DARPP-32, ARPP-21, D1 type receptors (D1R), D2 type receptors (D2R), encephalin (ENK) and dynorphin (DYN) were measured by RT-qPCR after 24 h treatment with BDNF or TSA. N = 5–8; +/− SEM (*p<0.05, **p<0.01, ***p<0.001).

We first examined the effect of HDAC inhibitors on survival and maturation of MSNs *in vitro*. Cells were treated with TSA (10 nM), VPA (3 mM) or compound 4 b (5 µM). There were no changes in neuronal death or survival after 24 h with any of the three HDAC inhibitors (Fig. 1a). HDAC inhibitors, therefore, do not increase survival of MSNs *in vitro* under these growth conditions.

We then surveyed the induction of MSN maturation by HDAC inhibitors, defined by level of DARPP-32 protein or mRNA. The largest increases in DARPP-32 were evident after 24 h of continuous treatment. Cells treated with TSA and VPA exhibited at least a doubling and sometimes almost a four-fold increase relative to control (Fig. 1b,c) whereas a two-fold increase was observed with compound 4b (Fig. 1d). Although morphologically homogeneous, MSNs are heterogeneous based on expression of neuropeptides and neurotransmitter receptors [20]. To determine whether all subtypes of MSNs respond to HDAC inhibitors, we assayed 1) Regulator of Calcium Signaling, which like DARPP-32 is a marker of all MSN subtypes [2]; 2) calbindin, a marker of the matrix compartment; 3) D1 type receptors (D1R) and dynorphin (DYN), markers of the direct pathway; and 4) D2 type receptors (D2R) and enkephalin (ENK), representative of the indirect pathway. Neurons treated with VPA for 24 h exhibited a two-fold increase in calbindin protein (Fig. 1e). The remaining markers were assayed by RT-qPCR and most were significantly increased by BDNF, TSA (Fig. 1f) and VPA (data not shown).

**Figure 2:**
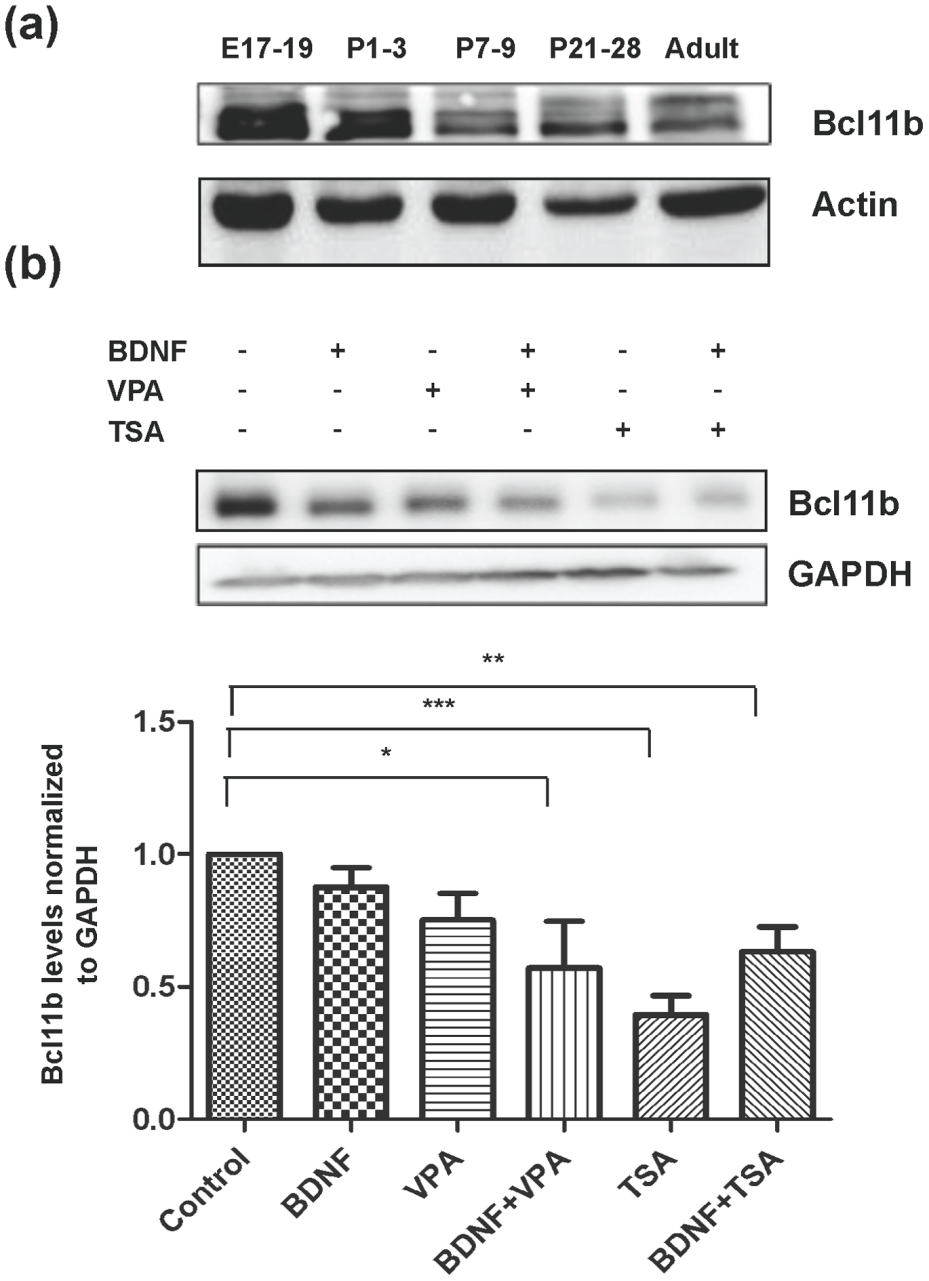
Bcl11b protein level is down-regulated in the presence of HDAC inhibitors. (**a**) Ontogeny of Bcl11b in striatum in Swiss-Webster mice. Bcl11b levels decrease markedly post-natally, particularly between the first and second week of life. (**b**) Primary striatal neurons were treated with VPA (3 mM) or TSA (10 nM) with or without BDNF (10 ng/ml) for 24 h. Similar results were observed after treatment with compound 4 b (not shown). N = 3–5; +/− SEM (*p<0.05, **p<0.01, ***p<0.001).

In addition to up-regulation of mature phenotypic markers, neuronal terminal differentiation is accompanied by repression of inhibitors of differentiation and genes required exclusively, or at higher levels, for earlier stages of maturation. All three requirements for differentiation may be regulated by HDAC inhibitors [21]. Bcl11b/CTIP2 is a transcription factor expressed in both striatum and cortex, and is required for development of striatal cellular architecture. *In vivo*, bcl11b level peaks during the late embryonic period, when it is expressed in both DARPP-32-immunopositive and immunonegative MSNs. Bcl11b protein is expressed in the adult striatum, but at a lower level than in the prenatal striatum, reaching adult level by postnatal day 7 (Fig. 2a), at which time DARPP-32 is still increasing [22], [23]. *In vitro*, bcl11b was significantly down-regulated when striatal precursors were cultured in the presence of TSA, consistent with its decrease during maturation of the striatum *in vivo*. There was also a downward trend with either BDNF or VPA alone, which became significant with the combination BDNF plus VPA (Fig. 2b).

### HDAC Inhibitors Increase Total Histone Acetylation in MSNs *in vitro*

**Figure 3:**
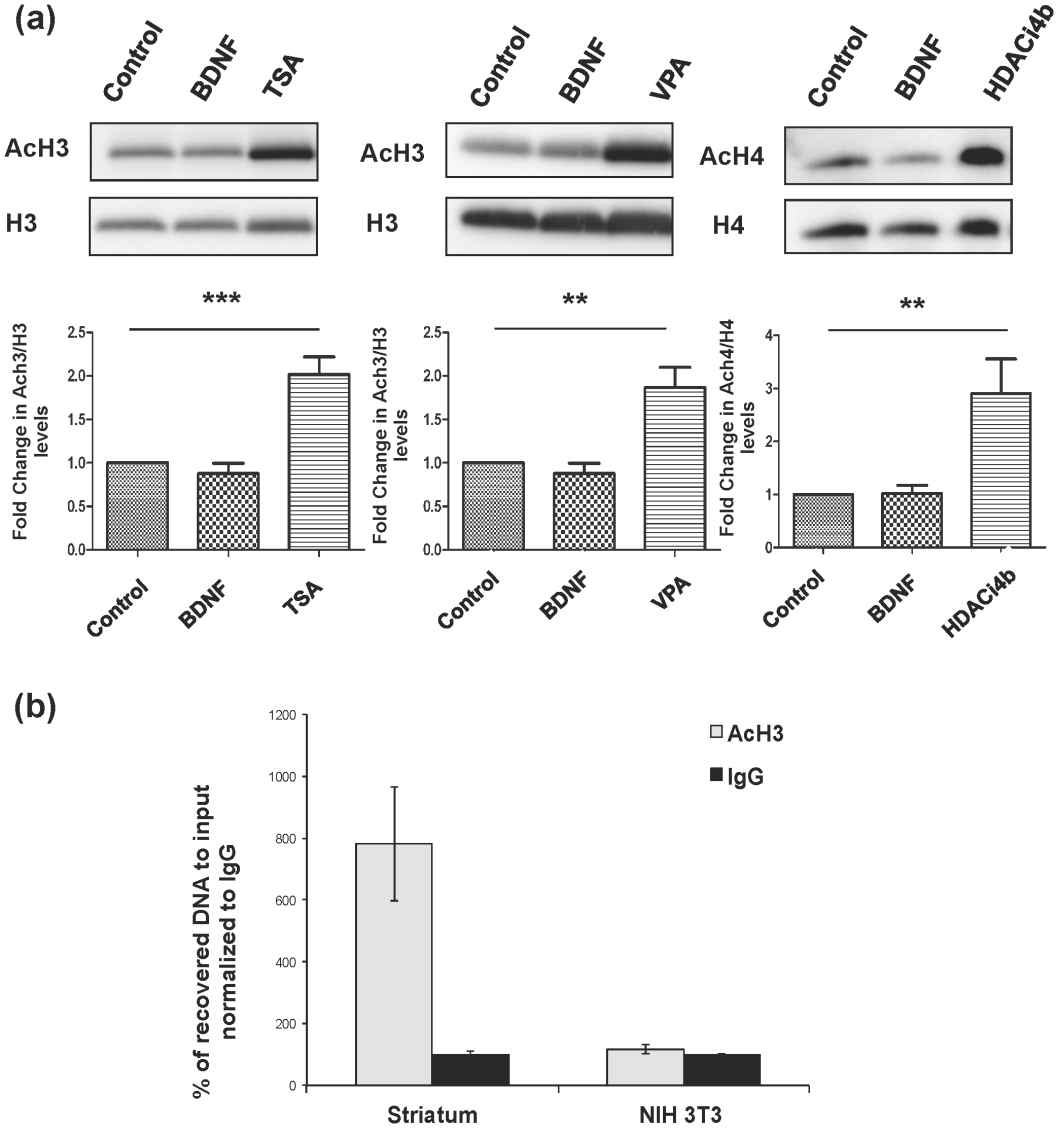
AcH3 is increased in striatal neurons by HDAC inhibitors and is enriched downstream of the transcription start site in *Ppp1r1b* striatal chromatin. (**a**) Cells were treated with BDNF (10 ng/mL), (a) trichostatin A (10 nM), (b) valproic acid (3 mM) or (c) HDACi 4b (5 µM) for 3 h. N = 3−5, +/− SEM (*p<0.05, ***p<0.001). (**b**) Chromatin was purified from adult mouse striatal tissue (STR) (DARPP-32-positive) and NIH-3T3 cells (DARPP-32-negative). Chromatin immunoprecipitation coupled with real-time PCR showed an increased association of acetylated histone H3 within a 1 kb region downstream of the DARPP-32 transcriptional start site (TSS) in striata relative to NIH 3T3 cells. Results are representative of two separate experiments performed in triplicates. Values for IgG are set at 100%. Error bars indicate SEM (*p<0.05).

We assayed the total level of acH3 and acH4 in MSNs *in vitro* after three hours (h) of treatment with TSA (10 nM), VPA (3 mM), or HDACi 4b (5 µM). As anticipated, there was an increase in acH3 with TSA and VPA, and an increase in acH4 with HDACi 4b (Fig. 3a). In fact, increased acetylation of both H3 and H4 was identified in the presence of all three compounds (data not shown). Importantly, exposure of plated cells to BDNF (10 ng/mL) for 3 hours did not produce an overall increase in H3 or H4 acetylation (Fig. 3a). To determine if HDAC inhibitors induce DARPP-32 via chromatin modification, we used chromatin immunoprecipitation (ChIP) RT-qPCR to assess the *in vivo* association of acetylated histone H3 (acH3) with ppp1r1b chromatin from DARPP-32-positive striatum and from DARPP-32-negative NIH 3T3 cells We focused on 1 kb upstream and downstream of the transcription start sites, utilizing commercial primer sets (SABioscience). Relative to NIH 3T3 cells, in which *ppp1r1b* is not transcribed, quantification indicated an approximately 7.8-fold (*p<0.05) enrichment of associated acH3 within 1 kb downstream of the DARPP-32 transcription start site (TSS) in the adult mouse striatum (Fig. 3b). Enrichment was not observed within 1 kb upstream of the TSS (data not shown). Despite a definite trend, we were unable to consistently demonstrate an enrichment of associated acH3 within 1 kb downstream of the DARPP-32 transcription start site (TSS) following HDACi treatment of cultured MSNs. Therefore, we are unable to conclude that HDACi’s induce DARPP-32 via chromatin modification. We do not, however, exclude that it occurs, as only a minority of the neurons express DARPP-32 even after treatment with an HDACi, leading to dilution of the chromatin of interest, and we did not survey the entire gene.

### HDAC Inhibitors Reduce Induction of DARPP-32 by BDNF

**Figure 4:**
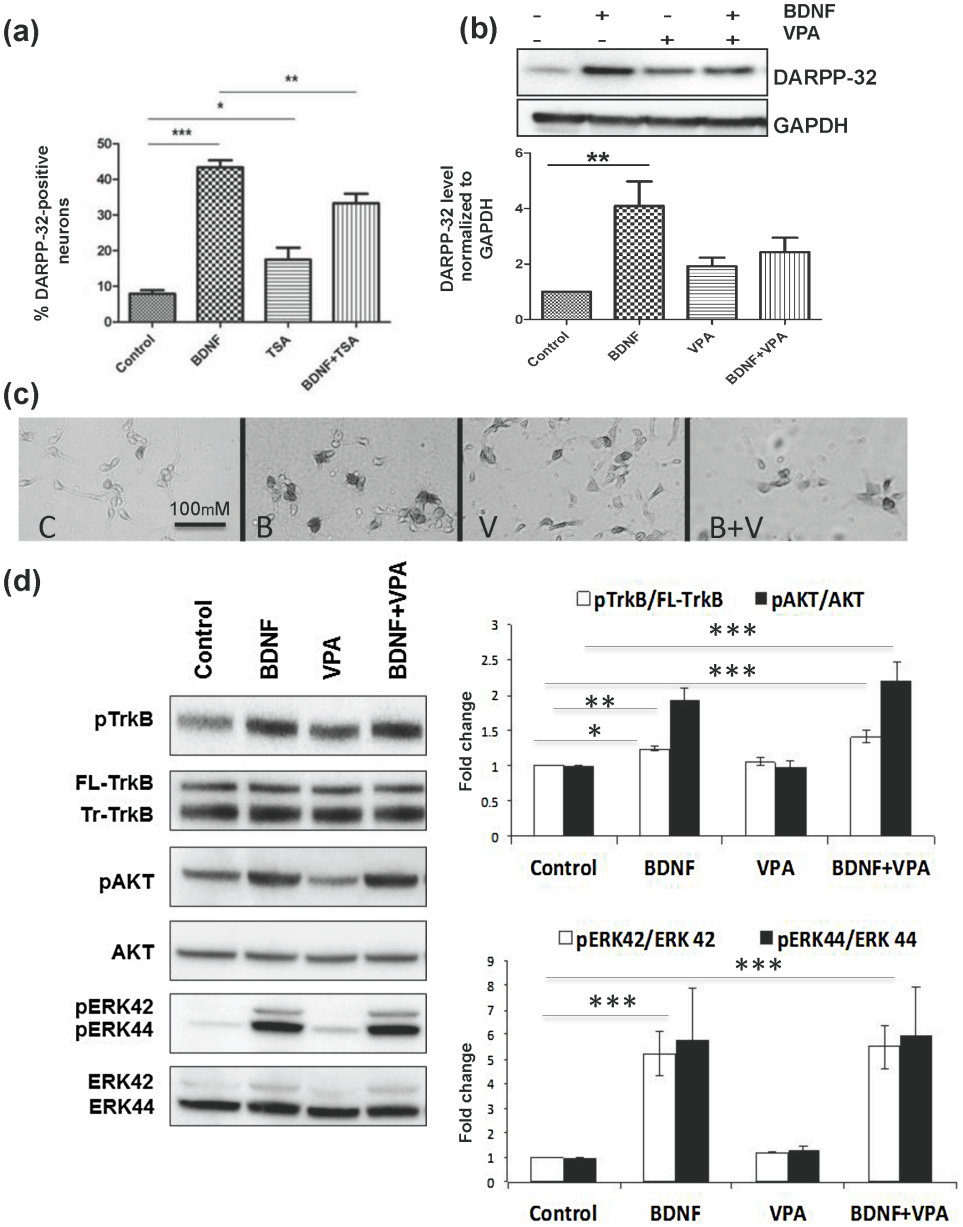
TSA and VPA inhibit induction of DARPP-32 by BDNF but do not inhibit the phosphorylation of TrkB, Akt or ERK in striatal neurons by BDNF. (**a**) Primary striatal neurons were treated with 10 ng/mL BDNF, 10 nM TSA, or BDNF plus TSA for 24 h. Cells were immunostained with α-DARPP-32. Immunopositive and total cells (>2,000 total) from 5 random fields from each treatment from 3 separate platings were counted and expressed as a percentage. One-way ANOVA with Newman-Keuls multiple comparison test was used for statistical analysis, (*p<0.05, **p<0.01, ***p<0.001). (**b**) Cells were exposed to a lower concentration of BDNF (5 ng/mL), VPA (3 mM), or BDNF (5 ng/mL) plus VPA (3 mM) for 24 h. N = 3−5; +/− SEM (*p<0.05, **p<0.01, ***p<0.001). (**c**) Representative photos of cells immunostained for DARPP-32 from experiment (b). There is a decreased number of immunopositive neurons in BDNF+VPA relative to BDNF only, and the neurons in BDNF+VPA also appear more lightly stained than in BDNF only; C = control, vehicle only; B = BDNF (5 ng/ml); V = VPA (3 mM). (**d**) Primary striatal neurons were treated with BDNF (10 ng/mL), VPA (3 mM), or BDNF plus VPA for 15 min. VPA (3 mM) treatment for 15 min did not induce phosphorylation of TrkB, Akt or ERK, nor did it inhibit the BDNF-induced increase in phosphorylation of all three signal transduction molecules. N = 3−5; +/− SEM (*p<0.05, **p<0.01, ***p<0.001).

We then sought to determine whether induction of DARPP-32 by BDNF and HDAC inhibitors is additive, similar to the effect on down-regulation of bcl11b. We treated cells with BDNF (10 ng/mL) alone, TSA (10 nM) alone and BDNF plus TSA for 24 h in 8-well slides and counted DARPP-32 immunopositive and immunonegative neurons. Surprisingly, the number of DARPP-32-immunopositive neurons was decreased after treatment with a combination of TSA and BDNF relative to BDNF alone (Fig. 4a). The percentage of DARPP-32-immunopositive neurons was 8% in control cultures, 17% with TSA only, 43% with BDNF only, and 33% with TSA plus BDNF. The differences were more apparent with western blotting following exposure to a lower concentration of BDNF. BDNF at 10 ng/ml may maximally up-regulate DARPP-32 expression, thereby masking an additive effect of an HDAC inhibitor. Based on previously published dose-response studies [3], we treated the neurons with 5 ng/ml of BDNF, with and without VPA. DARPP-32 level in cells treated with both 5 ng/mL BDNF and VPA was decreased by almost 50 percent relative to BDNF alone (Fig. 4b). Representative images of BDNF- and VPA-treated neurons, alone and in combination, immunostained for DARPP-32 are shown in Fig. 4c. We did not quantitate the level of immunostaining, but the relative intensities appeared lower, suggesting that the amount of DARPP-32 per cell was also decreased following treatment with both BDNF and TSA.

### HDAC Inhibitors do not Induce Phosphorylation of TrkB, Akt, or ERK in MSNs *in vitro*

VPA has multiple activities in addition to HDAC inhibition and chromatin modification, and activates many of the same signal transduction pathways as does BDNF [24]–[25]. Induction of DARPP-32 by BDNF requires TrkB, PI3K and Akt [4]–[6]. Induction of calbindin by BDNF is ERK dependent [26]. As we were unable to demonstrate *ppp1r1b* chromatin modification following HDACi treatment, we assayed activation of these pathways in primary striatal neurons following treatment with BDNF, TSA, VPA or compound HDACi4b for 15 min. None except BDNF led to phosphorylation of Akt (not shown and Fig. 4d). The remaining experiments were performed only with VPA as it is used in combination with BDNF in almost all protocols in which MSNs are derived from induced pluripotent stem cells (iPSCs) or neuronal stem cells (NSCs).

**Figure 5:**
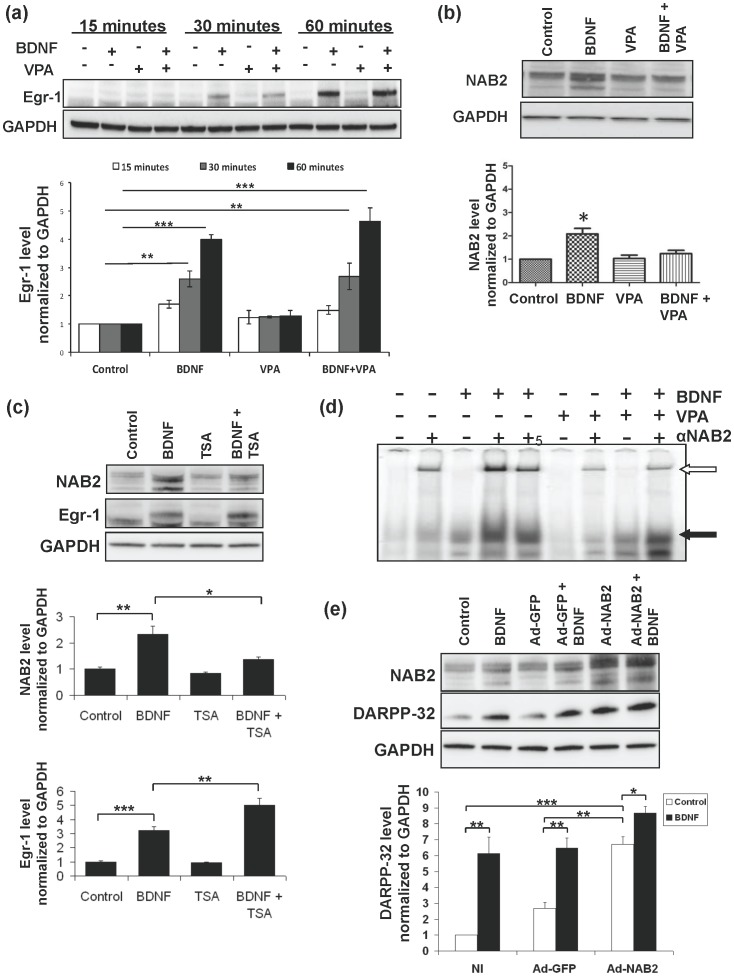
VPA and TSA do not inhibit induction of Egr-1 by BDNF, but do inhibit induction of Nab2 by BDNF. (**a**) Primary striatal neurons were treated with BDNF (10 ng/ml), VPA (3 mM), or BDNF (10 ng/ml) plus VPA (3 mM) for 15, 30, and 60 min. The level of Egr-1 was determined by western blotting. Results representative of at least 2 separate platings, performed in triplicate. Error bars indicate SEM (*p<0.05, **p<0.01, ***p<0.001). (**b**) Primary striatal neurons were treated with BDNF (10 ng/ml), VPA (3 mM), or BDNF (10 ng/ml) plus VPA (3 mM) for 3 hours and the level of Nab2 was determined by western blotting. Results representative of at least 2 separate platings, performed in triplicate. Error bars indicate SEM (*p<0.05). (**c**) Primary striatal neurons were treated with BDNF (10 ng/ml), TSA (10 nM), or BDNF (10 ng/ml) plus TSA (10 nM) for 3 hours. Levels of Egr-1 and Nab2 were determined by Western blotting. Results representative of 3 separate platings performed in duplicate. Error bars indicate SEM (*p<0.05, **p<0.01, ***p<0.001). (**d**) Primary striatal cultures were treated as in (b) followed by gel shift analysis. Anti-Nab2, 2 µg, was added before addition of DNA except in Lane 5, where it was added after the labeled oligonucleotide. Representative of 2 experiments performed in duplicate. The band representing binding of Egr-1 [7] is represented by the filled arrow, and the super-shift band generated by addition of α-Nab2 is indicated by the unfilled arrow. (**e**) Primary striatal cultures were treated with vehicle (NI:non-infected), Ad-GFP (Adenovirus – GFP), or Ad-Nab2 (Adenovirus – Nab2) with and without BDNF, as described in Methods. Representative of 3 platings, performed in duplicate. Error bars indicate SEM (*p<0.05, **p<0.01, ***p<0.001).

VPA did not lead to phosphorylation of TrkB, Akt or ERK, and did not inhibit BDNF-induced phosphorylation of TrkB, Akt or ERK (Fig. 4d). Induction of DARPP-32 by BDNF *in vitro* also requires an increase in Egr-1, aka NGF1-A, which binds to a sequence contained in a conserved region of the DARPP-32 gene to which we refer as H10 [7]. Induction of Egr-1 by BDNF plus VPA was equivalent to that of BDNF alone (Fig. 5a), leading us to investigate whether DARPP-32 induction by Egr-1 might require a co-activator or down-regulation of a co-repressor, particularly a member of the NGF1-A binding protein (NAB) family [27]. The Nab proteins, Nab1 and Nab2, derive their names from their original identification as molecules that are induced by nerve growth factor (NGF), bind to Egr-1/NGF1-A as it binds to chromatin, and act as co-repressors of Egr-1. Subsequently, their induction by multiple differentiation factors has been demonstrated, and they have also been shown to be capable of acting as co-activators of Egr-1-mediated transcription. Neither Nab1 nor Nab2 has been previously associated with BDNF. The level of Nab1 did not differ between control and treated neurons (data not shown), but Nab2 was markedly induced by BDNF at 3 hours, but not by VPA or importantly, by BDNF and VPA combined (Fig. 5b). Similar results were observed following treatment with TSA alone and with BDNF and TSA combined (Fig. 5c). The mechanisms of induction of Nab2 by BDNF and inhibition of its induction by VPA and TSA remain to be determined. Analysis of the gel shift patterns, previously confirmed as specific including the super shift produced by α-Egr-1 antibody [7], demonstrated that the band representing binding of Egr-1 was not increased between control and VPA (Fig. 5d, filled arrow), consistent with the lack of induction of Egr-1 by VPA; 2) the Egr-1 band was increased by BDNF alone and similarly by BDNF plus VPA; and 3) although an entire band was not super-shifted, addition of α-Nab2 antibody either before or after addition of the labeled oligonucleotide produced a super-shift that appeared greater in neurons treated with BDNF only (Fig. 5d, unfilled arrow; representative of 3 experiments). To directly investigate and better quantitate the effect of Nab2 in this system, we infected primary neurons with wild type Nab2. We found that Nab2 alone induced DARPP-32 expression, which was further increased by the addition of BDNF (Fig. 5e). We conclude that a minimal level of Nab2 is required for maximal induction of DARPP-32 by BDNF.

## Discussion

We examined the effects of HDAC inhibitors, TSA, VPA and HDACi4b, on the phenotypic maturation of MSNs *in vitro,* both alone and in combination with BDNF. HDCAi’s, particularly VPA, and BDNF are utilized for induction of mature neuronal phenotypes from iPS and ES cells, and are candidate treatments for HD [17], [28], [29]. Our main findings include: 1) TSA, VPA and HDACi4b increase the overall level of histone acetylation in MSNs; 2) acetylated H3 histone association is relatively enriched within 1 Kb downstream of the *ppp1r1b* TSS in striatal chromatin *in vivo*, but we were unable to demonstrate chromatin modification following *in vitro* treatment of MSNs with HDACi’s; 3) induction of DARPP-32 and other markers of the mature MSN by HDAC inhibitors does not require phosphorylation of Akt or ERK; and 4) BDNF induces Nab2, which is required for maximal induction of DARPP-32, and the induction of which is inhibited by VPA and TSA.

The data presented herein do not demonstrate that HDACi’s induce DARPP-32 via chromatin remodeling and in fact, do not definitively identify the mechanism via which this induction occurs. We previously identified two groups of transcription start sites in the *ppp1r1b* gene. One is grouped 400 ntd 5′ to the translation initiation codon, and the second 200–300 ntd further upstream. We also showed that the sequences both 1 Kb downstream and upstream of the TSS are unable to direct transgene expression to MSNs *in vivo* [30], [31]. Therefore, the hyperacetylation of the 1 Kb downstream of the TSS relative to the non-expressing NIH-3T3 cells is likely marking the core promoter region, i.e. an area of active transcription, but not necessarily a cell-specific enhancer [32]. Enrichment of DARPP-32-positive neurons will be required for a definitive study of the effects of HDACi’s and BDNF on acetylation of the *ppp1r1b* promoter region. It has recently been reported that many striatal-enriched genes are characterized by relatively increased H3 acetylation in coding portions rather than in the promoter, and that this correlation persists in the presence of mutant huntingtin and HDAC inhibitors [33]. Moreover, Egr-1 can modify acetylation status around its binding sites [34]. This may also contribute to our inability to consistently demonstrate an increase in bound acH3 after HDACi treatment around the promoter, and it will be important in the future to survey the entire *ppp1r1b* gene in a setting of enrichment for DARPP-32-positive neurons.

*In vivo*, striatal BDNF is largely derived via anterograde transport from the cortex, and only late death of MSNs in aged mice occurs following prenatal knockout of cortical BDNF [35]. BDNF is, however, required for the maturation of MSNs *in vivo* as demonstrated by the absence of calbindin and the delayed appearance of DARPP-32 in the BDNF-null striatum [36], [37] and the presence of abnormal dendritic and spine growth in MSNs in mice with a conditional forebrain deletion [38]. In most studies, exogenous BDNF does not have an impact on MSN survival *in vitro* [3], [39], [40].

BDNF requires Egr-1 for induction of DARPP-32 [7]. Nerve growth factor (NGF) and Egr-1 induce Nab2 in certain contexts [41], [42], but this is the first report of induction of Nab2 by BDNF. Originally identified as an Egr-1 co-repressor, it was later shown that Nab2 can also act as an Egr-1 co-activator [27], a function which is also context specific. It is possible, therefore, that the interaction between Egr-1 and Nab2 could serve to mediate both up-regulation and down-regulation of genes that occur during MSN differentiation. Moreover, and not surprisingly, it highlights the complicated pathways via which a growth factor and chromatin modifying agent alter expression level of a specific gene. It is consistent with the notion that induction of DARPP-32 by HDACi’s may not include increased acetylation at specific sites within the gene and may be indirect [33].

These data may be applicable to the goal of converting hES and hIPS cells to MSNs. The most widely used differentiation program to date is that of Aubry et al [14] in which neural precursors are treated with BDNF, and then with BDNF and VPA for terminal differentiation into MSN-like neurons. It is possible, therefore, that a combination of BDNF and VPA is actually inhibiting aspects of differentiation, particularly if DARPP-32 is used as the marker. A more robust differentiation protocol was recently reported in which VPA is added first in isolation, and is removed when BDNF is added days later [43], but perhaps the most robust terminal differentiation occurs in the presence of BDNF without addition of VPA [44]. Going forward, therefore, it will be prudent to assay multiple MSN markers in order to label the neuron as terminally differentiated. Similar issues are relevant to the search for HD treatments that do not include cell replacement. Significant effort is being expended on identification of agents to pharmacologically manipulate the BDNF/TrkB pathway and HDAC activity. As with most neurodegenerative diseases, poly-pharmacy will likely be required [45], highlighting the need to determine interactions between potential treatments.
